# Characterizing the benthic community in Maryland’s offshore wind energy areas using a towed camera sled: Developing a method to reduce the effort of image analysis and community description

**DOI:** 10.1371/journal.pone.0215966

**Published:** 2019-05-02

**Authors:** Wilmelie Cruz-Marrero, Daniel W. Cullen, Najja R. Gay, Bradley G. Stevens

**Affiliations:** 1 Living Marine Resources Cooperative Science Center, Department of Natural Sciences, University of Maryland Eastern Shore, Princess Anne, Maryland, United States of America; 2 Biology Department, Lawrence University, Appleton, Wisconsin, United States of America; Universidad de Cádiz, Facultad de Ciencias del Mar y Ambientales, SPAIN

## Abstract

Offshore wind farms are a crucial component for the improvement of renewable energy in the United States. The Bureau of Ocean Energy Management (BOEM) designated ~170 km^2^ of shelf area for wind energy development off the coast of Maryland, USA. In order to understand potential environmental impacts of wind turbine installation on the benthic ecosystem within the designated area, we conducted a study to visually characterize bottom habitats and epibenthic communities in the Mid-Atlantic Outer Continental Shelf blocks of the Maryland wind energy area. Seven 5 km long transects were sampled using a towed camera sled with a downward-facing digital camera that captured images at 5 frames·s^-1^s. Additional small-mesh beam trawling was also conducted at selected locations complementary for species identification. Image data were analyzed using two image selection methods, random and systematic (i.e. video frames were selected at various intervals). For both methods, estimates of community diversity (Hill’s N2) stabilized with sample sizes ranging from 316 to 398 frames. Our results allowed us to define distinct epibenthic communities and bottom habitats that are associated with offshore wind energy sites and to develop a sampling technique for digital images that can be applied to other research programs.

## Introduction

The construction of offshore wind turbines may significantly impact Mid-Atlantic benthic ecosystems. For example, wind farms have caused changes in commercial vessel routes [1], fish communities [2], and marine mammal foraging behavior [3]. Additionally, the presence of wind farms in certain areas may result in losses of habitat for some sea birds and disrupt marine mammal migration patterns [4,5]. Other known effects on the environment include wave transformations [6], and electromagnetic impacts [4]. Conversely, in addition to providing an alternative energy source and reducing greenhouse gas emissions, the base of windmill, the cable and associated rocks around the structures may serve as artificial reefs, and could enhance the settlement of aquatic species both native and non-native species [4,7,8,9,10].

**Fig 1:**
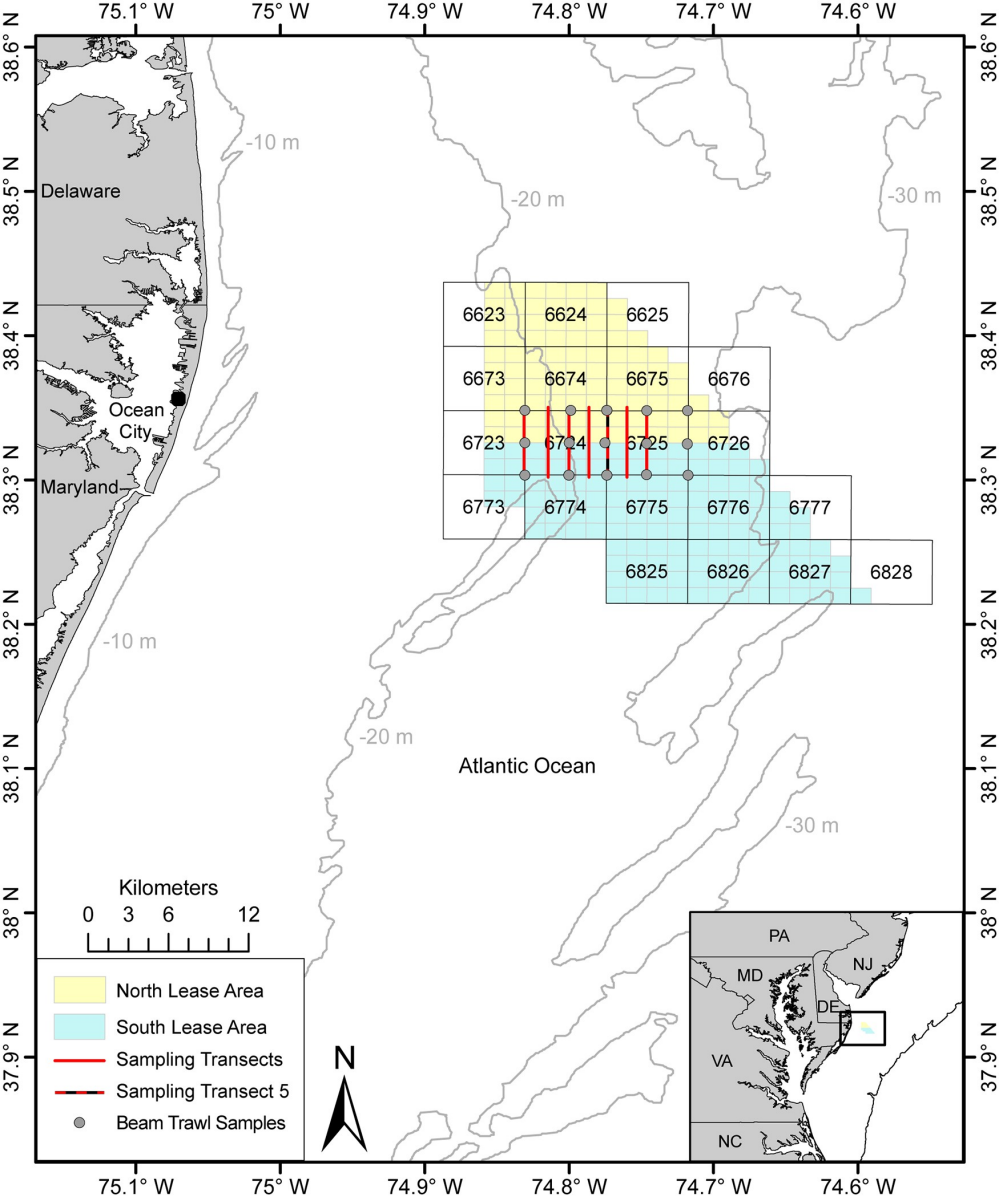
Map of Outer Continental Shelf blocks in the Maryland Wind Energy Area (WEA). The large blocks are statistical areas and the small yellow and green squares indicate the north and south lease areas, respectively. The grey circles indicate the locations of beam trawl samples (BT01-BT15). The solid red lines indicate the sampling transects within the WEA; the red and black line indicates the sampling test set from Transect 5. The inset shows the location of the WEA off the Maryland Coast.

The United States (US) consumes 18% of the world’s primary energy [11]. About 11% of the energy comes from renewable sources (e.g. geothermal, solar, wind, hydroelectric) [11]. In 2010, the US Department of Energy created the initiative “Smart from the Start” which involved the development of renewable wind energy on the Atlantic Outer Continental Shelf (OCS) [12, 13]. The OCS has been divided into 3 x 3 nautical mile (nmi) blocks, each of which includes an area of 2304 hectares. Groups of these OCS blocks have been identified as high priority wind energy areas (WEAs) in the states of New Jersey, Delaware, Maryland, and Virginia [12]. The OCS seafloor is distinctive for its scarcity of structures and predominance of soft sediments such as sand, silt and clay [14,15]. The focus of the current study was in the ~170 km^2^ Maryland section of the WEAs [12]. The Maryland WEA is divided into northern and southern regions and encompasses a total of nine full lease blocks and eleven partial blocks [16]. Approximately 85% of commercial fishing conflicts lies in the southern areas close to block 6774 in the Maryland WEA [17]. However, the southern area contains documented slow-growing cold-water coral species, and portions of the area have been used as commercial or recreational fishing grounds [17] (Fig 1). Species captured in the WEAs during Northeast Fisheries Science Center (NEFSC) fall trawl surveys include economically and ecologically important fish and invertebrate species including black sea bass (*Centropristis striata*), goosefish (*Lophius americanus*), bluefish (*Pomatomus saltatrix*), weakfish (*Cynoscion regalis*), scup (*Stenotomus chrysops*), windowpane flounder (*Scophthalmus aquosus*), summer flounder (*Paralichthys dentatus*), cancer crabs (i.e. Jonah crab *Cancer borealis*, rock crab *Cancer irroratus*), American Lobster (*Homarus americanus*) and other species [18]. Understanding the long term impacts of wind energy turbine installation requires knowledge about bottom sediments and habitat types as well as fish and benthic assemblages. This information is currently lacking for most of the WEAs.

Our sampling was primarily conducted using a camera sled (hereafter referred to as CamSled). The CamSled is an innovative system by which both organisms and ecosystems can be quantified. Sampling with underwater camera gears allows an investigator to make direct observations of organisms and their behavior including interactions with other species. Additionally, underwater camera gears provide *in situ* images of the sea floor and habitat structure [19]. Compared to methods that utilize SCUBA such as underwater visual census, the use of a camera sled is not limited by sampling depth or bottom time [20,21,22]. Additionally, the field of view for a camera used with a camera sled is fixed allowing for a standardized view of bottom sediments and habitats. Furthermore, geographic locations of images are captured within the frames, and can be used for habitat mapping [23,24,25].

The goal of this project was to conduct a survey of benthic habitats and biota in order to provide data that can be used to understand potential environmental impacts of the wind turbine installation in the Maryland WEA. Specific objectives of this study were: 1) sample and characterize benthic habitats and species diversity within the Maryland WEA region and 2) develop a sampling technique to adequately estimate species diversity most efficiently from our underwater image data.

## Materials and methods

Scientific collection permit for this research was granted from the Maryland Department of Natural Resources Fisheries Service in 2014 under Permit Number SCP201406.

### Sampling with camera sled

The Maryland WEA, located ~20 km off the coast of Ocean City, MD [12], is divided into north and south lease areas that were previously divided into blocks (Fig 1). Nine Transects were initially defined within OCS blocks 6724 and 6725, which are between the northern and southern areas. Seven of the initial nine transects were sampled due to time constraints (Fig 1). Transects were 5 km long (~60–90 minutes per transect), oriented along a North-South axis, and were spaced at intervals of 1.25 km.

**Fig 2:**
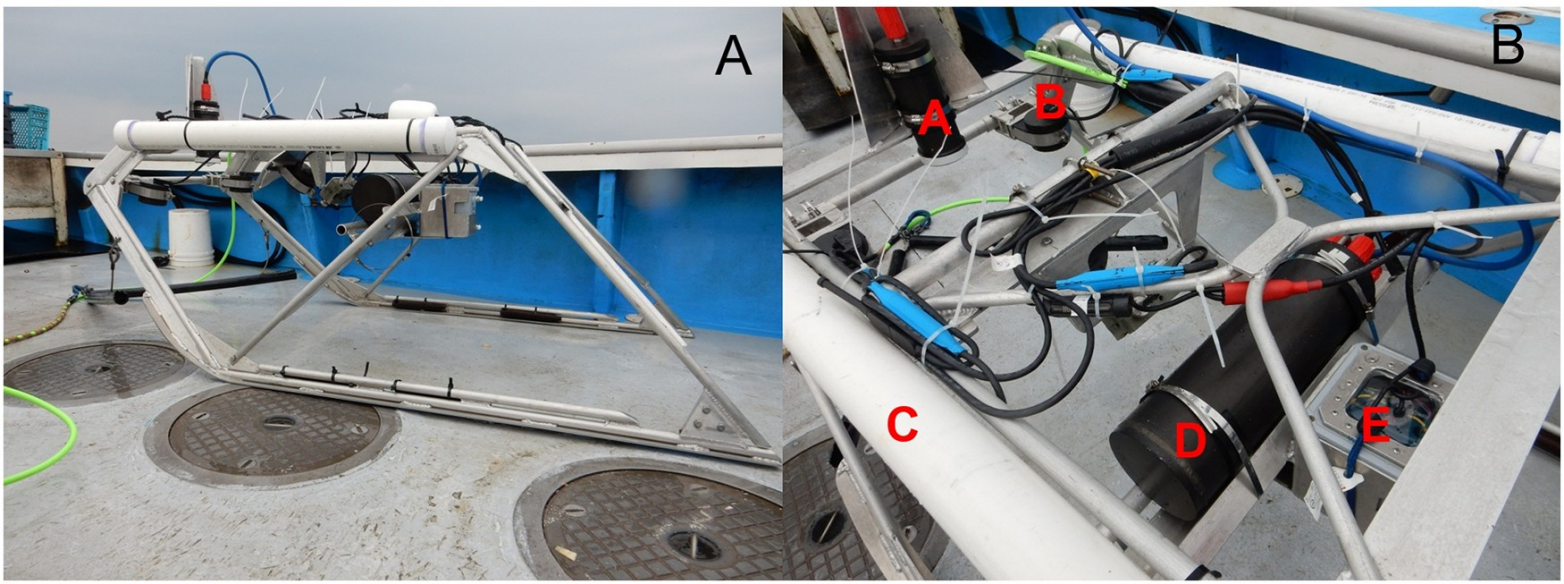
(A) Camera sled used to sample fish and invertebrate species from 1 June 2014 to 30 October 2014 in the offshore wind energy area located off the coast of Maryland, USA. (B) Physical components of the camera sled including (a) camera, (b) strobe lights, c) floats, (d) on-board computer, and (e) battery.

The CamSled (dimensions: ~ 2 m long × 1 m wide × 1.5 m high) was constructed of aluminum round and flat bar and could be easily disassembled for transport (Mark Blakeslee, AquaLife, Kodiak AK, aqualife@ak.net) (Fig 2A). The CamSled had a camera (Point Grey Research, Inc. Zebra2 5.0 MP 2448 x 2048 at 25 FPS with High Definition-Serial Digital Interface, Sony ICX625 CCD) in a vertical position, facing directly downward with a field of view of approximately 1.1 m wide. Additionally, three synchronized strobes were added to the sled to enhance light and resolution of the images (Rick Towler, Fisheries Research Instrumentation, Seattle WA, rtowler@gmail.com). Each strobe is comprised of four Bridgelux BXRA-C2002 LED arrays driven at ~30 Watts each for a total of 120 Watts/strobe. Each LED array emit ~2400 lumen per array which equals to 9600 lumen per strobe. Strobes housing was designed by Mac Marine Instruments with a depth rating of 1000 meters.

**Fig 3:**
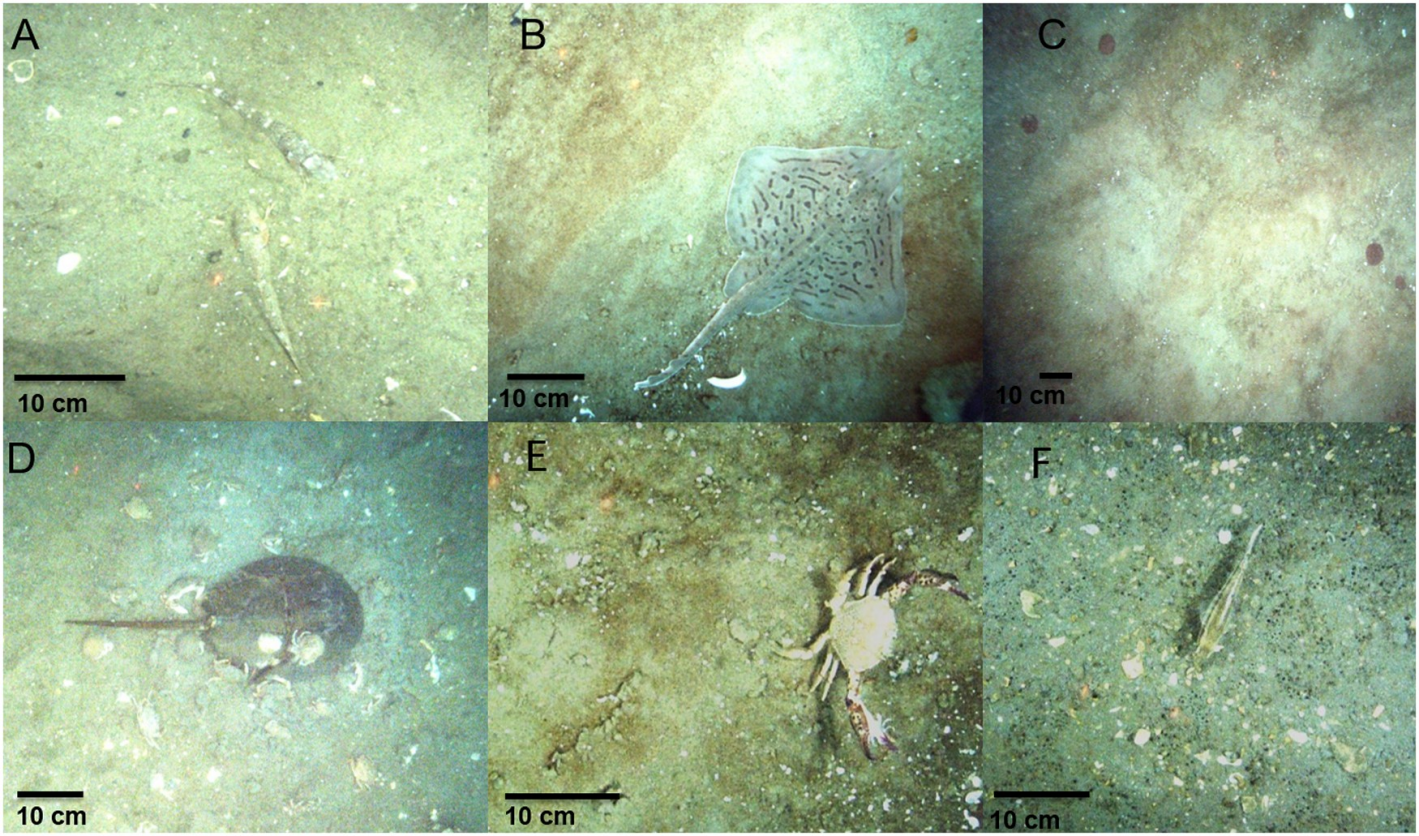
Example of video frames collected with the camera sled while sampling fish and invertebrate species from 1 July 2014 to 30 March 2015 in the offshore energy wind area located off the coast of Maryland, USA. A) Northern sea robin, B) Clearnose skate, C) Sand dollars, D) Horseshoe crab, cancer crabs, and long-clawed hermit crabs, E) Lady crab; F) Black sea bass. The red dots are laser beams separated by a distance of 10 cm, used for measuring fish and invertebrates in images.

During sampling, the CamSled was towed from a chartered commercial vessel with a boom and hydraulic block, and was weighted to insure that it stayed on the bottom. The Camsled was towed at the slowest speed achievable by the vessel, typically 2–3 knots (1–1.5 m.sec^-1)^. During transect tows, a computer (with a hard drive) on board the CamSled provided real-time communication with a laptop on the vessel which allowed us to control the operation of the camera (Fig 2B). Using the NorPix Streampix 6 software application, high definition digital images were recorded directly to a hard drive on board the CamSled at 5 frames∙s^-1^ at an exposure of 10 ms. Images had an approximate overlap of 25% so that any feature or organism on the seafloor showed up in 3−5 consecutive frames. At such high shutter speeds, motion is stopped, and high resolution images are captured that allow easy identification of species with a resolution of about 2 cm (Fig 3). The positions of the vessel during each transect were acquired with a Garmin GA 38 Global Positioning System (GPS) antenna, and position of the CamSled was fixed by adding or subtracting its layback from the vessel position. Sled layback (L) was calculated within each transect, assuming the cable catenary was a straight line, [26] using the formula:

$$L=\surd (R^{2}-D^{2})$$

where (L) is the distance from the boat to the CamSled, (R) is the length of the tow cable, and (D) is depth of each transect. Since all tows were conducted in a northern or southern direction, layback was either subtracted or added, respectively, to the vessel’s latitude to determine the correct position of the CamSled.

### Image analysis

To reduce the image annotating process for the CamSled survey, we developed a sampling technique to determine the diversity index of species encountered during transects and to reduce the effort required for the annotation and analysis of our video images. Because each organism appeared in 3−5 images due the fast shutter rate, we developed an efficient method to reduce the frame overlap and to sample the data without analyzing all the available images.

A test set comprising a total of 9155 sequential frames or 30 consecutive minutes of a single transect was examined using images from Transect 5 (hereafter referred to as test set) (Fig 1). Within the test set, all of the observed organisms (>2.5 cm) were identified to the nearest taxonomic level, and counted within each frame. Sediment types were subjectively classified as silt, sand, shells, boulders and clay (i.e. rare compacted dark sediment). Sediments were annotated using visual estimation of percentage composition charts from Terry et al. [27].

After analyzing all video images from the test set, and in order to test the feasibility of sampling in 15 minute segments data from the test set were divided in half with each half (designated 5A and 5B) consisting of 4577 frames.

From the test set data, we selected subsamples using both a systematic and random sampling design. For the systematic samples, 40 different subsamples were defined by systematically selecting frames at multiple intervals ranging from 5 to 300 frames; this yielded sample sizes ranging from 30 to 1831 frames, constituting 0.3% to 20% of the test set. Initial starting frames for each sample were randomly selected before conducting systematic samples to reduce any bias. For the random samples, 36 different subsamples were defined by randomly selecting sample sizes that increased from 31 to 1778 frames in equally spaced logarithmic intervals, constituting 0.3% to 19% of the test set. Once we determined the best sampling technique using the test set, R software was used to select images from every 30^th^ frame from all the transects. A total of 4393 frames were annotated in seven transects. Equivalent to the test set data, all of the observed organisms were identified to the nearest taxonomic level and sediment was classified using the same technique mentioned above.

### Beam trawl sampling

Beam trawl sampling was conducted with a small mesh net in order to verify species identifications from the video count data. The beam trawl (4.66 m × 1.83 m with a 5 mm liner; 1.27 cm mesh net) was attached to an aluminum frame. Tows were conducted for 10 min at 15 stations (referred hereafter as stations BT-1 through BT-15) distributed evenly throughout the two OCS blocks 6724 and 6725 (Fig 1). Stations were located at the north ends, south ends, and midpoints of Transects 1, 3, 5, 7, and 9 (Transect 9 was not sampled with the CamSled) (Fig 1). At a nominal speed of 1 m sec^-1^, the beam trawl sampled an area of approximately 1098 m^2^ (1.83m x 600sec) or almost twice the area sampled by a Camsled tow of the same distance. The total area sampled by the beam trawl was therefore determined to be about 16,500 m^2^. All specimens caught were counted and identified to the lowest possible taxonomic group. One large catch of >3600 sand dollars (*Echinarachnius parma)* was subdivided into 6 approximately equal trays; one tray was fully counted, and the total catch was estimated by multiplying the value of the counted tray by six.

### Data analysis

Because of variations in tow speeds, currents, and wind conditions, it was difficult to accurately estimate the distance of the area surveyed by both the beam trawl and the CamSled. For those reasons, species density and abundance could not be estimated precisely from our data so we chose to estimate diversity as an indicator of epibenthic community composition and condition.

Diversity indices for both CamSled data and beam trawl were calculated from the proportion of total organisms (p) in each taxon using Shannon’s H’ [the uncertainty of species identification; H = -sum(p*log(p))], and Hill’s N2 [the effective number of abundant species; N2 = 1/sum(p^2^)] [28]. For the test set, locally weighted regression scatterplot smoothing (LOWESS) was used to examine the relationship between Hill’s N2 and the log10-transformed sample size (number of images) for both systematic and random sampling. Means of the diversity indices of each repetition and the coefficient of variation (CV) were calculated for all the data sets.

### Cluster analysis (CamSled and beam trawl)

Organism counts were compared between transects using hierarchical cluster analysis. Prior to conducting cluster analysis, data (counts) were centered (i.e. each was expressed as a residual from the overall mean) and scaled (i.e. to units of standard deviation). A distance matrix was calculated using Euclidean distance, and the cluster analysis was conducted using Ward’s minimum variance method.

## Results

### Image analysis and frame counts

**Fig 4:**
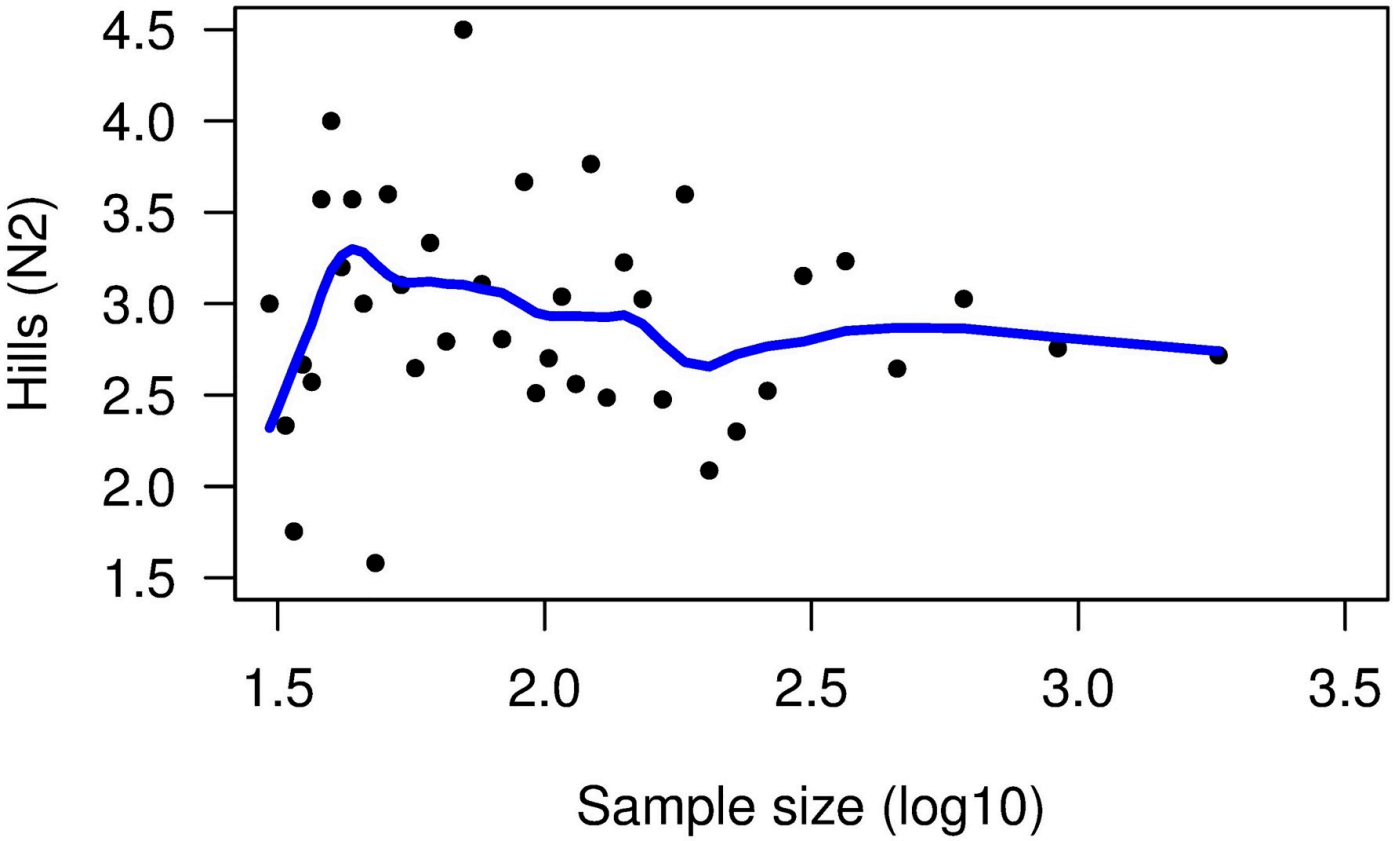
Systematic sampling of the test set. Hill’s N2 Diversity index is plotted against the log10-transformed number of frames sampled. The blue line is the LOWESS (locally weighted) regression line that levels out around 10^2.6^ (398 frames).

**Fig 5:**
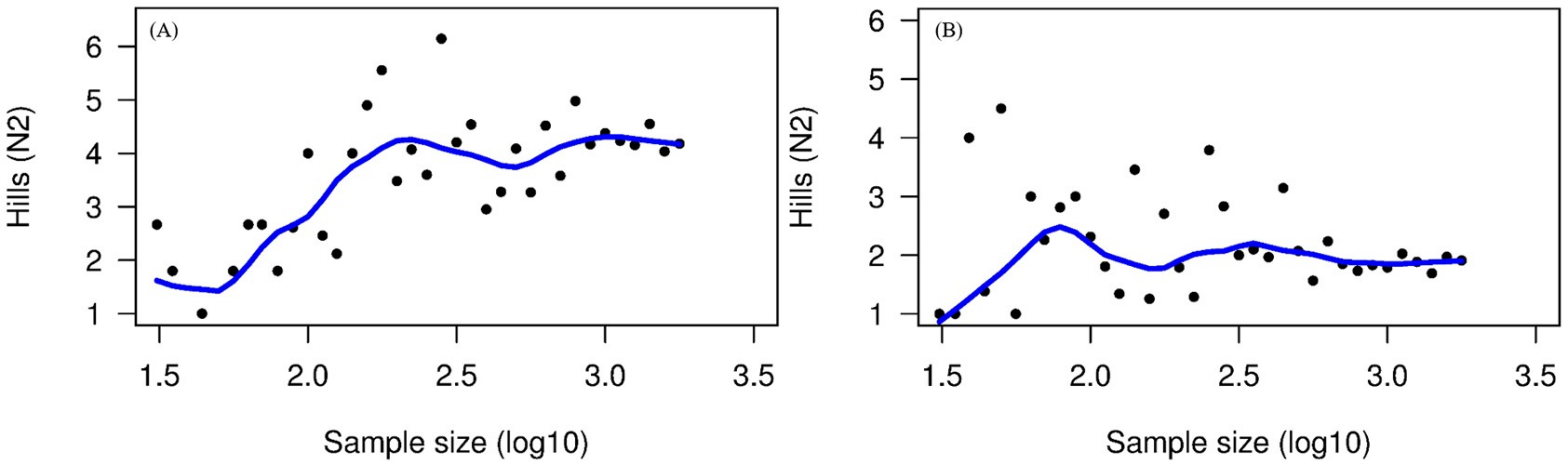
Random sampling of the first and second halves (15 min segments) of the test set. Hill’s N2 Diversity index is plotted against the log10-transformed number of frames sampled. Only one of 10 trials is shown for each half-transect. The blue line is the LOWESS (locally weighted) regression line. Diversity leveled out at sample sizes around 10^2.5^ (316 frames) in (A), and at about 10^2.6^ (398 frames) in (B).

**Fig 6:**
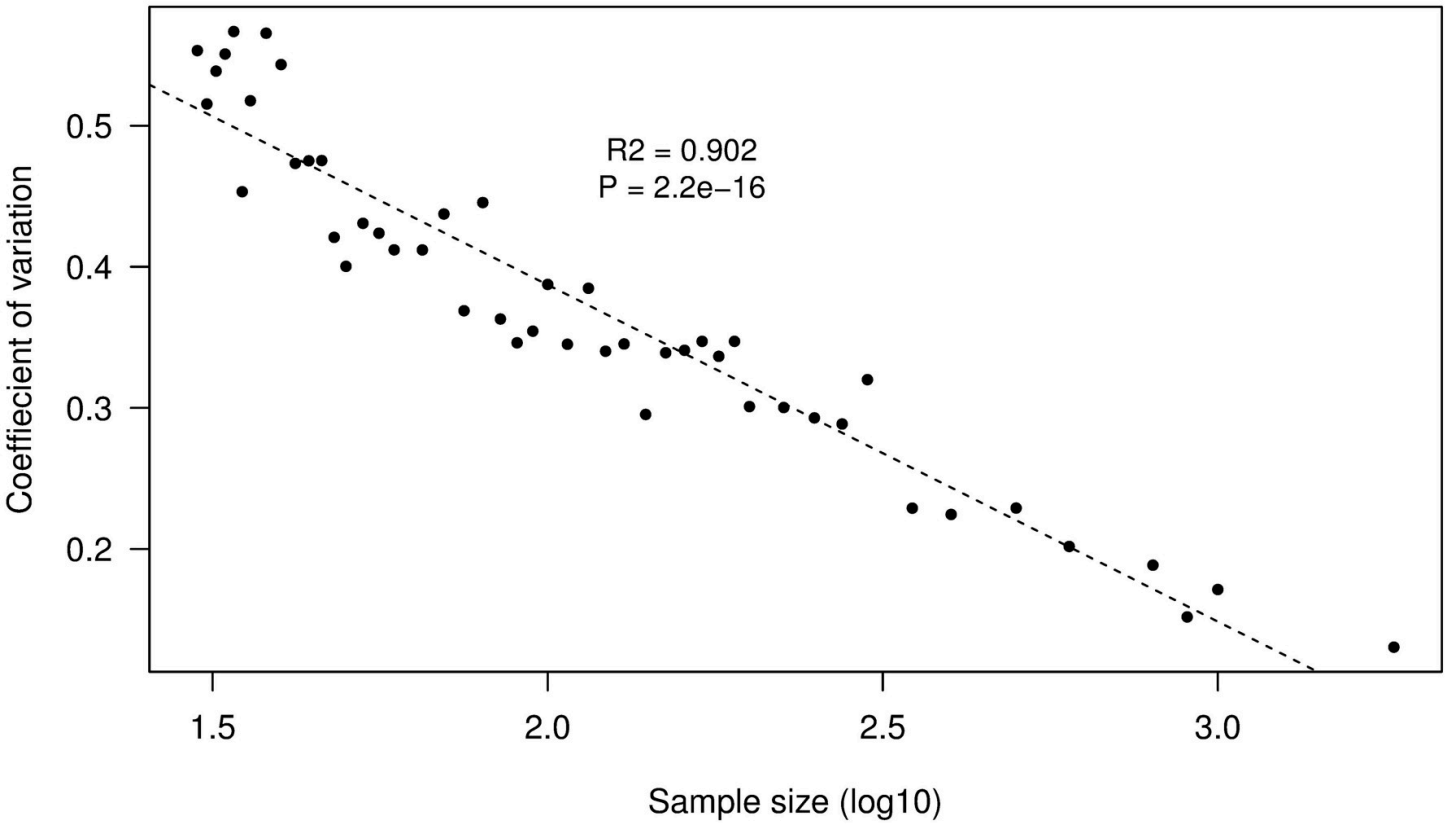
The relationship between the coefficient of variation of the different sampling sizes using the random technique (R^2^ = 0.902) and log10 transformed sample size.

A total of 9155 frames were examined in the test set. In the complete test set, we observed 339 organisms representing 13 species in 27% of the frames (73% of the frames contained no organisms). The systematic sampling technique showed that diversity estimates varied greatly at sample sizes <100 frames, but stabilized (i.e. approached the value for the completely analyzed sample) at a sample size of approximately 398 frames and a sample interval of 30 frames (Fig 4). Diversity estimates from random sampling of frames leveled off at sample sizes of n = 316 frames in the first half of the test set (Fig 5A), and at a sample size of n = 398 frames in the second half (Fig 5B), corresponding to sampling intervals of 24 to 30 frames, respectively. Repeated sampling by this method produced similar results, although the pattern of variation at small sample sizes differed between them. We calculated the coefficient of variation (CV) and standard deviation of all the repetitions to obtain the amount of variability between the means (Fig 6). We determined that an acceptable coefficient of variation for the annotation of the test set was 25%. This level of variation occurred at sample sizes similar to those at which the LOWESS regression leveled off, i.e ~316 frames or 10^2.5^.

Based on the results of systematic and random sampling methods, we concluded that there was no clear difference between random and systematic samples, and that unbiased estimates of diversity could be obtained by sampling at intervals ranging from 24 (random) to 30 (systematic) frames. Because systematic sampling was much easier than random sampling, data from all transects were sampled systematically at intervals of 30 frames.

The total number of usable frames counted was 4393. Some CamSled transects had incomplete coverage due to problems with the sled hardware or software, or poor water visibility. As a result, numbers of usable frames per transect varied from 34 to 1036 (Table 1). Since the usable frames may have been scattered throughout the samples, seafloor area surveyed is not directly compatible to the beam trawl. Organisms were observed in 1463 of the 4393 frames examined (33.3%), whereas the majority of frames (2930; 66.6%) were empty. A total of 3035 individual organisms representing 23 taxonomic groups were counted and grouped into seven taxonomic classes. We were able to identify some organisms to the species level (e.g. sand dollars), whereas others could only be identified to higher taxonomic levels (e.g. Brachyura or Actiniaria) (Table 2). Because the level of identification varied, most analyses were conducted on data at either the level of class or order to avoid comparing numbers between different taxonomic levels.

**Table 1:** Mean values of depth (m), frames counted using the 30^th^ technique, Shannon’s H’, and Hill’s N2 diversity indices using organisms identified to the class level for all the observations and for each Transect.

Data	Depth (m)	Frames	Shannon H	Hill’s N2
Trans1	15.1	563	1.072	2.072
Trans2	22.8	34	0.974	2.462
Trans3	24.4	806	1.223	2.560
Trans4	24.4	35	0.970	2.419
Trans5	26.5	1036	0.587	1.357
Trans6	25.8	1007	0.780	1.559
Trans7	26.8	912	0.576	1.361
All	24.5	4393	1.266	2.917

The most common species observed by the CamSled samples were plain sand dollar (*Echinarachnius parma*), hermit crab (*Paguridae* spp.; present in 9.3% of examined frames), sand lance (*Ammodytes americanus*), chestnut astarte clam (*Astarte castanea*), and Forbes seastar (*Asterias forbesi*) (Table 2). Echinoderms (e.g. sand dollars) occurred most frequently in Transects 1 and 5, whereas hermit crabs were most abundant in Transects 5, 6 and 7. Values of H’ and N2 for the complete data set were 1.74 and 3.208, respectively, when organisms were analyzed at the order level and 1.266 and 2.917, respectively, when analyzed at the class level (Table 1). This indicates that the number of dominant species (~3) did not change with the level of analysis because they were all in different orders (i.e. sand dollars, hermit crabs, and sand lance). When analyzed at the class level for individual Transects, diversity (Hill’s N2) was greatest for Transects 1−4, where it ranged from 2.07 to 2.56, and lowest for Transects 5–7 (range 1.36 to 1.56) (Table 1).

**Table 2:** Total numbers of organisms observed in beam trawl samples, CamSled samples from the test set and all organisms observed in subsampled frames from Transects 1−7. Organisms are arranged in descending order.

Common name	Scientific name	Beam trawl	Test set	Transect 1–7
plain sand dollar	*Echinarachnius parma*	6961	10	1294
hermit crab sp.	*Pagurus sp*.	464	249	882
auger snail	*Terebra dislocata*	296	0	0
american sand lance	*Ammodytes americanus*	29	0	231
forbes sea star	*Asterias forbesi*	165	48	85
chestnut astarte clam	*Astarte castanea*	29	0	213
long-clawed hermit	*Pagurus longicarpus*	0	0	159
rock crab	*Cancer irroratus*	134	0	4
gulf stream flounder	*Citharichthys arctifrons*	133	5	1
northern sea robin	*Prionotus carolinus*	30	5	52
moon snail	*Lunatia sp*.	12	0	63
warty nudibranch	*Onchidoris sp*.	61	0	14
spotted hake	*Urophycis regia*	50	0	0
sand shrimp	*Crangon septemspinosa*	43	0	0
unknown cancer crab	*Cancer sp*.	0	1	13
anemone	*Anemone unknown*	0	13	5
lady crab	*Ovalipes ocellatus*	5	0	0
sea cucumber	*Pentamera sp*.	5	0	0
clearnose skate	*Raja eglanteria*	0	0	4
sea whip	*Leptogorgia virgulata*	0	1	4
lined sea horse	*Hippocampus erectus*	3	0	0
surf clam	*Spisula solidissima*	3	0	0
unknown bony fish	*Osteichthyes sp*.	0	0	3
windowpane flounder	*Scophthalmus aquosus*	2	3	1
black sea bass	*Centropristis striata*	0	0	2
four spot flounder	*Paralichthys oblongus*	2	0	0
horseshoe crab	*Limulus polyphemus*	0	2	2
northern pipefish	*Syngnathus fuscus*	2	0	0
squid	*Loligo or Illex sp*.	2	1	0
channeled whelk	*Busycotypus canaliculatus*	0	1	1
left-eyed flounder	*Bothidae sp*.	0	0	1
unknown crabs	*Crustacea sp*.	0	1	1
winter skate	*Leucoraja ocellata*	1	0	0
unknown polychaete	*Polychaete sp*.	0	3	0
	Total:	8432	343	3035

**Fig 7:**
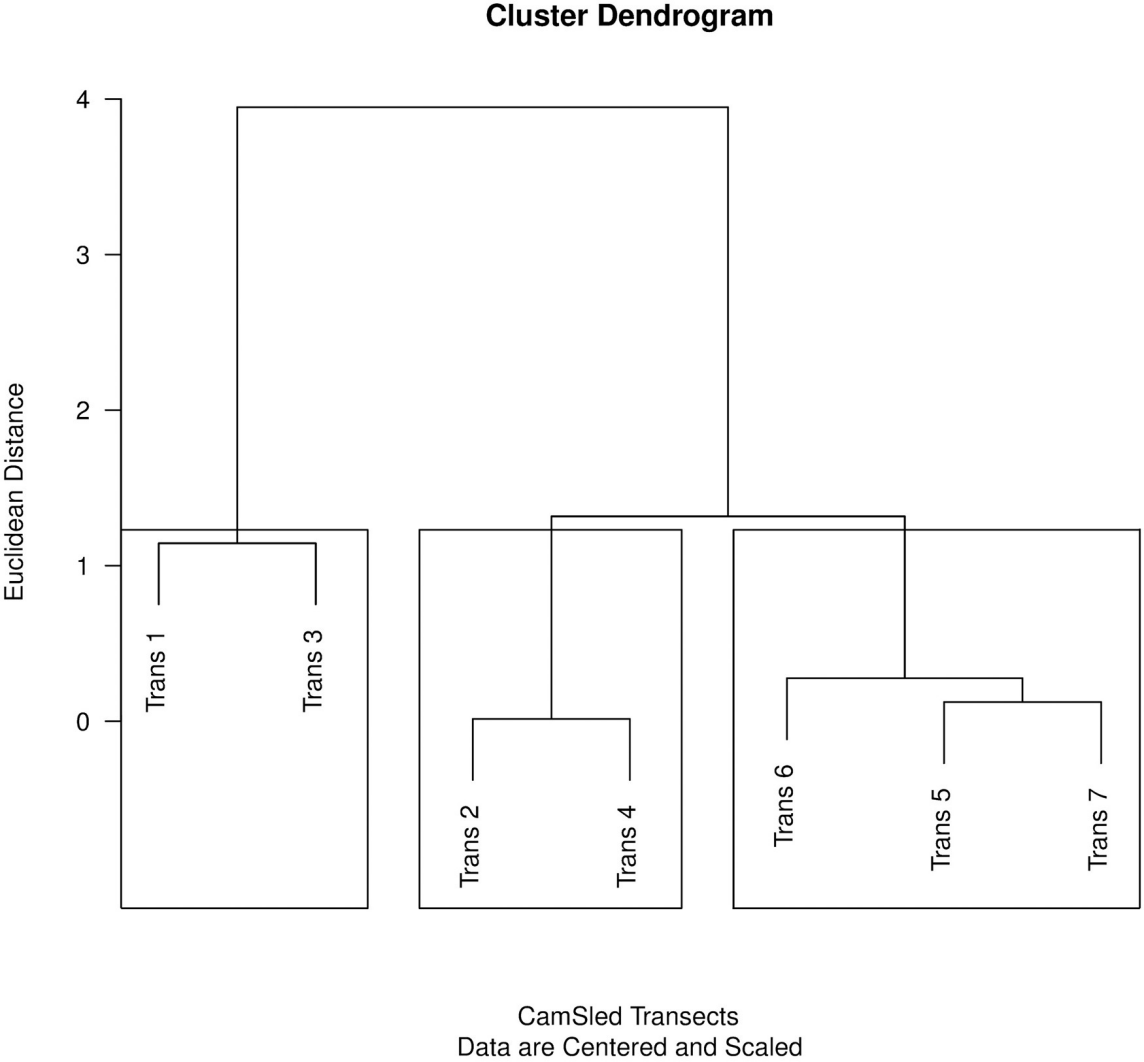
Dendrogram showing the distribution of species from the camera sled counts. Counts were centered and scaled before analysis. Three clusters were identified based on organism class identification. The cluster containing Transects 5−7 was the most completely sampled, but also had lower diversity.

**Fig 8:**
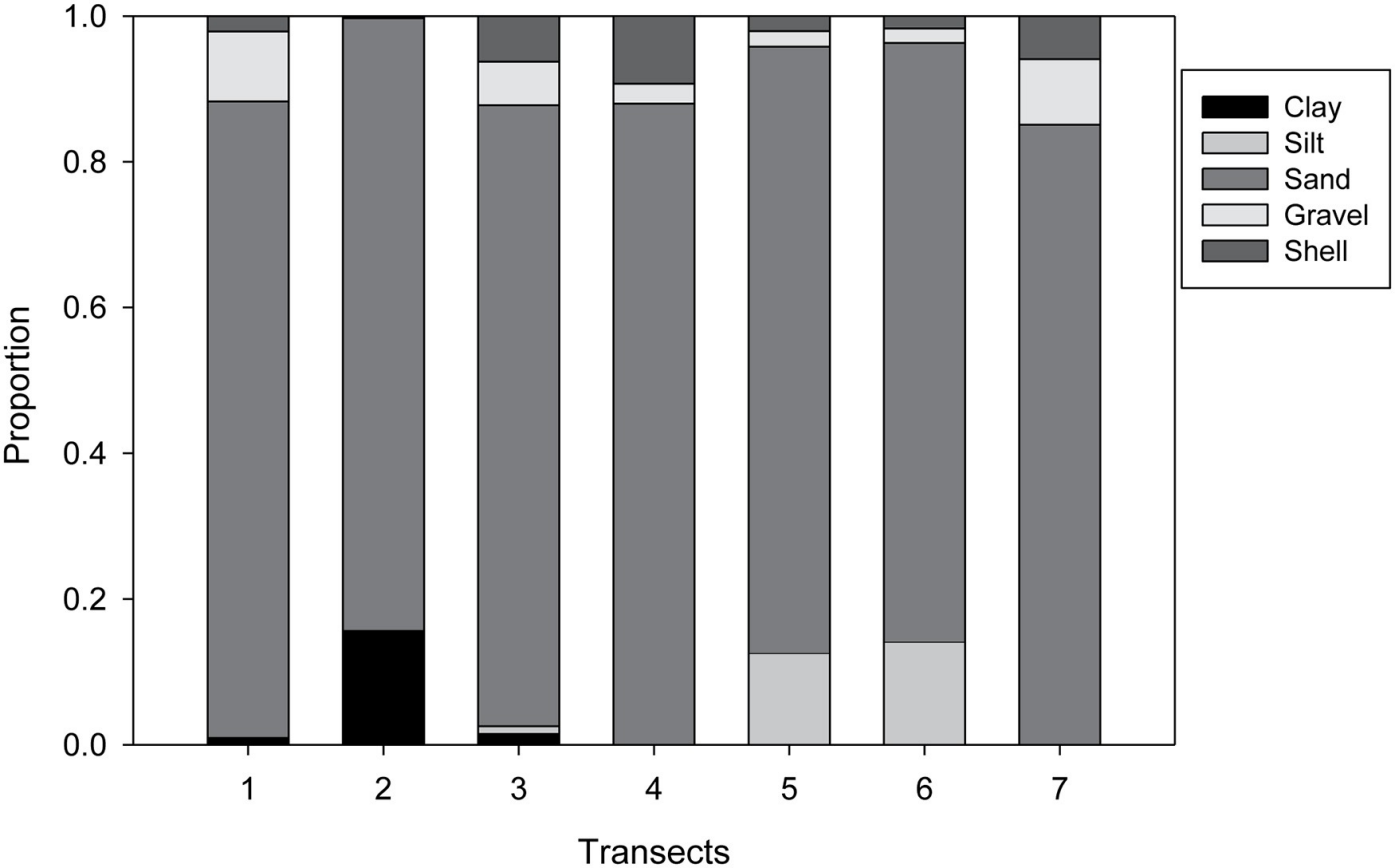
Proportions of sediments observed within CamSled Transects. Sediments were classified as clay, silt, sand, gravel and shell.

Video frame counts of organisms were compared between Transects using cluster analysis. The cluster dendrogram indicated three clusters of Transects (Fig 7). Transects 1 and 3 were most similar to each other, Transects 2 and 4 were similar, and Transects 5, 6, and 7 were similar. Mean depth for all Transects was 24.5 m (± 0.12), with a range from 15.1 to 26.8 m. The distribution of observed substrate types among transects varied from sand to gravel with sand being the most common substrate observed (Fig 8) Transects 5 and 6 had more silt than other Transects. There was little gravel or other hard substrata observed in any of the video frames. Cluster analysis of sediment types indicated that Transects fell into three groups. Transects 1, 3, 4 and 7 formed a single group. Sediments on Transect 2 appeared to consist of compacted clay, differing from previously defined sediment types. Transect 2 stood by itself, and Transects 5 and 6 formed the third group. Transect tended to fall into similar cluster groups, separated a long on the East-West gradient.

### Beam trawl sample analysis

**Fig 9:**
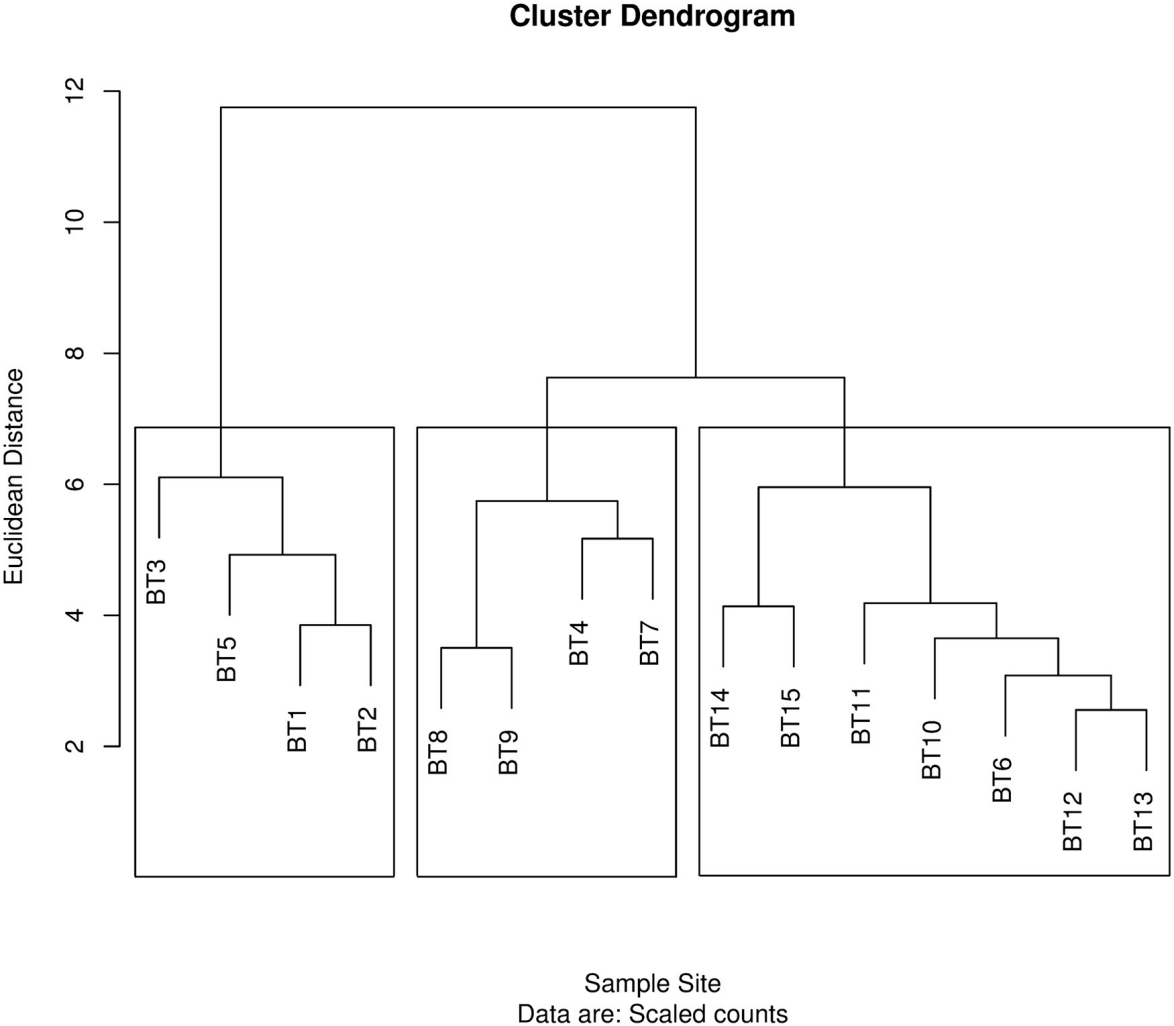
Dendrogram from cluster analysis of beam trawl samples based on species abundance. Hill’s N2 diversity index, which represents the effective number of abundant species in each cluster, was for 4.39 for box 1 2.40 for box 2, and 2.09 for box 3. Cluster 3 was dominated by sand dollars.

Beam trawl data were analyzed to determine species diversity and community structure. We captured 8432 organisms representing 22 species, of which 17 consisted in more than two individuals (Table 2). The most common organisms observed were: plain sand dollar (*Echinarachnius parma*), hermit crab sp. (*Pagurus sp*.), auger snail (*Terebra dislocata*), Forbes sea star (*Asterias forbesi*), rock crab (*Cancer irroratus*), and gulf stream flounder (*Citharichthys arctifrons*). A total of 6961 or 82.6% of plain sand dollar were observed, >3600 were caught in one tow at station BT-15. Cluster analysis was conducted using the 14 taxa with counts ≥ 5 individuals because species that are rarely observed would contribute little to diversity estimates. Cluster analysis defined 3 distinct benthic communities that were loosely organized along depth gradients (Fig 9). Overall mean values for the two diversity indices were 1.628, and 2.787 for Shannon’s H, and Hill’s N2. Sand dollars were the dominant species in cluster 3, comprising most of OCS block 6725. This resulted in a very low diversity in that block (Hill’s N2 = 2.09, Table 3).

**Table 3:** Depth and diversity of beam trawl sites (BT) 1–15. Shannon’s H’ and Hill’s N2 diversity indices were calculated using organisms identified to the nearest taxonomic level.

Transects	Depth (m)	Shannon	Hill’s N2
BT1	18.29	1.955	5.521
BT2	19.51	1.951	4.354
BT3	20.73	1.251	2.275
BT4	20.12	1.866	5.245
BT5	27.43	2.136	6.779
BT6	27.13	0.441	1.210
BT7	27.13	1.333	2.479
BT8	29.26	0.825	1.502
BT9	26.21	0.330	1.129
BT10	26.52	1.539	3.113
BT11	26.52	1.512	3.334
BT12	25.30	0.778	1.536
BT13	26.21	1.506	2.910
BT14	28.96	1.092	1.923
BT15	28.96	0.152	1.050
All		1.628	2.787

## Discussion

In the present study, estimates of epibenthic diversity produced by systematic sampling using a camera sled became consistent at a sampling interval of 30 frames, and at sampling intervals of 24 to 30 frames for random sampling. Based on these results, we concluded that unbiased estimates of diversity could be obtained by sampling all of the remaining image files at intervals of 30 frames. At a rate of 5 frames/s, the CamSled produces 300 photographic frames/min, or 18,000 frames per hour, and most Transects lasted from 60–90 min. When sampled at intervals of 30 frames, a one-hour Transect would produce 600 sampled frames. This meant examining 1 frame every 6 s. These results are likely specific to our data, since sampling intervals of 24 to 30 frames were derived from specific sample sizes of 316−398 frames. Different data sets might require smaller or larger samples, and consequently, different sampling intervals. Additionally, some sections of Transects were not photographed due to equipment failures, or were photographed but could not be counted due to poor image quality. This is the case for Transects 2 and 4 which produced a total of 974 and 1016 frames. Subsampling those transects at 30-frame intervals produced less than 316 frames, which could have affected the precision of diversity indices. Subsequent modifications have improved reliability of the CamSled considerably. Although time codes were recorded on the photographs taken for Transects 5−7, they were not recorded for Transects 1−4 due to technical issues. For this reason, we could not subdivide the Transects into smaller samples for comparison to beam trawl samples. Thus we concluded that estimates of organism abundance or density from our data would have been inaccurate.

Beam trawls have been reported by some authors to be more accurate than visual methods and other trawl types for the sampling and identification of small benthic and cryptic species [29, 30]. For example, Cailliet et al. [29] compared the use of trawls, camera sleds, and submersibles for sampling fish assemblages off the coast of California and found that beam trawls were more advantageous for sampling smaller fishes than the visual methods used. Similarly, Walsh and Guida [30] sampled fish and macroinvertebrate assemblages close to designated WEAs in Mid-Atlantic US continental shelf waters during the spring and found that beam trawls caught more benthic taxa and smaller individuals than bottom trawls. In our study, three species were among the top six in both the beam trawl and CamSled samples (sand dollars, hermit crabs, and seastars). These are similar to the major species in the benthos reported by the NEFSC [31], except that we did not see scallops in our survey. The beam trawl samples were examined closely so that small invertebrate and fish species could be identified, and many of these were < 5 cm, including nudibranchs, sand shrimp, and sea cucumbers, all of which were too small to identify to species in CamSled images. Additionally, though rare, squid were observed by both sampling gears. The beam trawl also dug into the sediment slightly, accounting for the presence of many small organisms, such as auger shells, shrimp, and nudibranchs that were less commonly observed in CamSled frames. The majority of auger shells identified in the beam trawl were inhabited by hermit crabs, so all similar shells were identified as hermit crabs in the CamSled frames. Although sand dollars were the most abundant species in the beam trawl samples, most of them came from station BT-15, and the remainder were mostly from stations BT-12 through BT-14, on Transects 7 and 9; Transects 8 and 9 were not sampled with the CamSled due to time constraints. Diversity indices were overall low for the CamSled (Shannon’s H 1.266, Hills 2.917), and beam trawl (Shannon’s H (1.628); and Hill’s N2 (2.787) which indicates accordance between the CamSled and trawl surveys. Only a few species were dominant and diversity of species was low for both techniques.

Within the area sampled, there was little variation in depth, except for Transect 1, which was considerably shallower than the others. Transects 2, 5, and 6 had slightly lower proportions of sand than other transects. There is a slight gradient in depth, sediment type, and community structure from east to west. We did observe some outcrops of clay similar to those described by Steimle and Zetlin [15], but sand was the dominant sediment type in the survey area as it is for most of the mid-Atlantic region. Both the beam trawl and CamSled surveys showed that diversity decreased from east to west, most likely due to the dominance of sand dollars in the western-most Transects. Thus, the area of our research (between north and south lease areas) was similar to the three blocks in the south lease areas that were surveyed in 2012, in terms of sediment type and lack of hard substrate [32]. Other studies in Europe that involved the sampling of hard substratum introduced during turbine construction have reported that it increased the number of species present and biodiversity [33,34]. The community of benthic organisms observed in the present study was made up primarily of semi-mobile invertebrates (sand dollars, clams), mobile invertebrates (crabs, snails, seastars), and highly mobile fish. Due to the lack of hard substrate encountered in the sampling area, very few sedentary organisms (i.e. those that need to attach to hard substrata) were observed, except for a few anemones and sea whips. The sediment types and homogeneous habitats we identified in this study are common for the Mid-Atlantic area [14]. Boulders and rock patches have been described as a habitat for sea whips in the mid-Atlantic but none were observed during this study [15].

Our results indicate that the epibenthic community of the study area can be characterized as one that is comprised of mobile species that are adapted to highly unstable substrata (sand). The US Mid-Atlantic continental shelf has been receptive to man-made structures such as buoys, shipwrecks and artificial reefs that have enhanced various fisheries [15]. The introduction of new hard substrata in this area could enhance the low species diversity of the survey region [9, 10]. For example, in the Baltic Sea, construction of wind turbines increased the abundance of sessile invertebrates in both the water column (attached to turbines) and the adjacent seafloor; more species of fish were found near the turbines, and abundance of schooling fish (primarily two-spotted gobies) increased by an order of magnitude relative to control sites [35]. Studies conducted before and after turbine construction at Horns Rev (Denmark) showed that the noise caused by the turbines did not seem to impact fish [2, 33]. In general, it has been documented that succession is a primary factor driving community diversity and composition at offshore wind farm locations [33,35,36,37]. The colonization of the pillars at Horns Rev started with filamentous algae, followed by the introduction of suspension feeders, sub-surface deposit feeders, and herbivores and carnivores. A similar successional pattern could occur in the Maryland WEAs. More specifically, the addition of hard bottom structures in the WEA would provide new surfaces for colonization by common Mid-Atlantic epibenthic species including bryozoans, hydroids, anemones, and stony and sea whip corals (*Leptogorgia* sp.) [15]. The novel habitats would also likely be utilized by decapod crustaceans and demersal fishes including economically important species such as American lobster (*Homarus americanus*), Jonah crab (*Cancer borealis*), black sea bass (*Centropristis striata)*, Tautog (*Tautog onitis*), and red hake (*Urophycis chuss*). Further, fish species commonly found near Mid-Atlantic reefs such as bluefish (*Pomatomus saltatrix*), grey triggerfish (*Balistes capriscus*), and summer flounder (*Paralichthys dentatus*) may use the structures for feeding or as a flow refuge. Finally, the hard surfaces may provide important attachment areas for squid (*Loligo* sp.) to deposit their eggs [15].

Both the CamSled and beam trawl gears had advantages and limitations for sampling the epibenthic community of the Maryland WEA. The beam trawl sampled ten species that were absent in CamSled samples while CamSled samples contained eight unique species. Sampling with the CamSled was more complex and difficult due to intermittent equipment failure and poor image sample quality. Further, the analysis of the photographic data set was labor-intensive although our image processing technique allowed us to reduce the time required for annotation. Conversely, although the beam trawl sampled smaller species more efficiently than the CamSled, trawl gears are known to have a significant impact on epibenthic habitats, organisms, and seafloor topographies [38, 39]. Lastly, though our second objective was to develop a sampling method to estimate species diversity adequately from the underwater image data, we did observe lower diversity estimates for species in transects (i.e. Transects 5, 6, and 7) that included more sampled video frames. We attribute this result to the high number of sand dollars we observed in those Transects. However, different diversity estimates may have been obtained if equal numbers of frames were sampled from all transects. Therefore, although we do believe that we developed an efficient technique for estimating diversity from our image data, the results from any future assessments utilizing image data collected with the CamSled would be improved with more equal sampling among transects.

The goal of our research was to assess the epibenthic community of the WEA and due to the limitations of both the camera sled and beam trawl, use of both gears produced complementary data for the assessment of the Maryland environment. Therefore, we conclude that both gear types should be used for future benthic assessments of the Maryland WEA. The data we gathered will be usable for studying the long term impacts of WEA development. This document provides background information usable for planning prior to construction or installation of wind turbines, and which can be used for comparison to future, post-construction surveys.
